# DPAFuse: A dual-space probabilistic adversarial image fusion framework with robust coding embedding

**DOI:** 10.1016/j.fmre.2026.03.024

**Published:** 2026-04-30

**Authors:** Hao Zhang, Meiqi Gong, Douyu Wu, Jiayi Ma

**Affiliations:** Electronic Information School, Wuhan University, Wuhan 430072, China

**Keywords:** Image fusion, Robust coding embedding, Probabilistic adversarial learning, Dual-space representation, Distribution consistency

## Abstract

Multimodal image fusion aims to integrate complementary information from heterogeneous sensors, yet existing methods remain vulnerable to composite degradations and inherent modality bias. On the one hand, representations become unreliable under composite degradations; on the other hand, fusion strategies often directly enforce cross-modal consistency, inevitably forcing the fused results into inter-modal compromises and leading to unstable representations. To address these issues, we propose DPAFuse, a dual-space probabilistic adversarial image fusion framework with robust coding embedding. Firstly, a robust coding embedding mechanism projects heterogeneous inputs into a unified latent space, contracting the distributional discrepancies among diverse composite degradations and aligning them closer to the underlying clean representation, while effectively suppressing degradation-related components and preserving modality-invariant structural and semantic features. Moreover, to alleviate modality bias, we design a dual-space probabilistic adversarial fusion mechanism that enforces distribution-level consistency across latent and image domains, enabling adaptive global fusion without local compromises. Extensive experiments on public datasets demonstrate that DPAFuse achieves superior robustness and fusion fidelity compared with state-of-the-art methods. The code is publicly available at https://github.com/HaoZhang1018/DPAFuse.

## Highlights

• Robust coding embedding extracts reliable features under composite degraded conditions.
• Dual-space adversarial fusion aligns distributions and preserves structures.
• DPAFuse achieves superior recovery and fusion in diverse degraded environments.

## Graphical abstract

**Figure:**
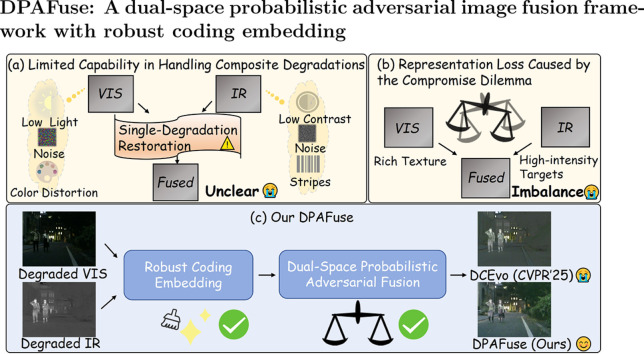

## 1 Introduction

Due to the inherent limitations of the imaging and perception of individual sensors, a single type of sensor can only provide partial information representation, making it insufficient to achieve comprehensive scene perception in complex environments. For example, visible-light imaging sensors share a perception mechanism similar to that of the human visual system and are capable of capturing rich texture and color information. However, their representational capability becomes limited under challenging conditions, such as poor illumination or occlusion. Against this background, multimodal image fusion [1], [2] has emerged as an effective solution that integrates complementary information from different sensors, thereby enhancing the robustness and accuracy of perception systems in complex environments. For instance, integrating visible-light imaging sensors with infrared imaging sensors enables the preservation of texture and color details from visible images, while leveraging the strong penetrative capability of infrared imaging under challenging conditions such as low illumination and occlusion, thereby achieving more stable and reliable scene perception across diverse scenarios. Benefiting from the superior representational capability of fused images, multimodal image fusion has been widely applied in various critical domains [3], [4], [5], [6] such as autonomous driving, intelligent surveillance, computer-aided diagnosis, military reconnaissance, and remote sensing monitoring, demonstrating both theoretical significance and practical value.

Multimodal image fusion has long been a research hotspot in computer vision. The paradigm of traditional fusion methods typically follows three stages: extraction, fusion, and reconstruction. First, features are extracted from multisource images using fixed mathematical transformations. Second, these multisource features are aggregated using simple fusion rules, such as addition, mean, or maximum. Finally, the corresponding inverse transformation is applied to convert the fused features back into a fused image. However, traditional approaches rely on hand-crafted transformations and simple fusion rules, thereby limiting their ability to model complex cross-modal relationships and often result in suboptimal performance under diverse imaging conditions [7], [8]. To alleviate these limitations, deep learning-based fusion methods have been introduced, which replace one or more stages of the conventional extraction-fusion-reconstruction paradigm with learnable neural networks and can therefore be broadly categorized into two types according to the stages they replace. Non-end-to-end methods such as DenseFuse [9] and DIDFuse [10] typically adopt an autoencoder architecture, where deep learning is mainly used for feature extraction and image reconstruction, while the fusion module itself still relies on handcrafted rules or manually designed criteria. In these methods, the encoder replaces traditional fixed transformations to extract more representative deep features, while the decoder reconstructs the fused image from the integrated feature representation. In contrast, end-to-end fusion methods typically integrate the above three stages into a single network. Methods such as U2Fusion [11] and CrossFuse [12] demonstrate that fully learnable architectures can jointly optimize feature extraction and fusion in a unified framework. The entire pipeline is jointly optimized using pixel-level distance losses or task-driven objectives, enabling more flexible and adaptive modeling of cross-modal relationships compared with manually designed fusion rules.

**Fig. 1:**
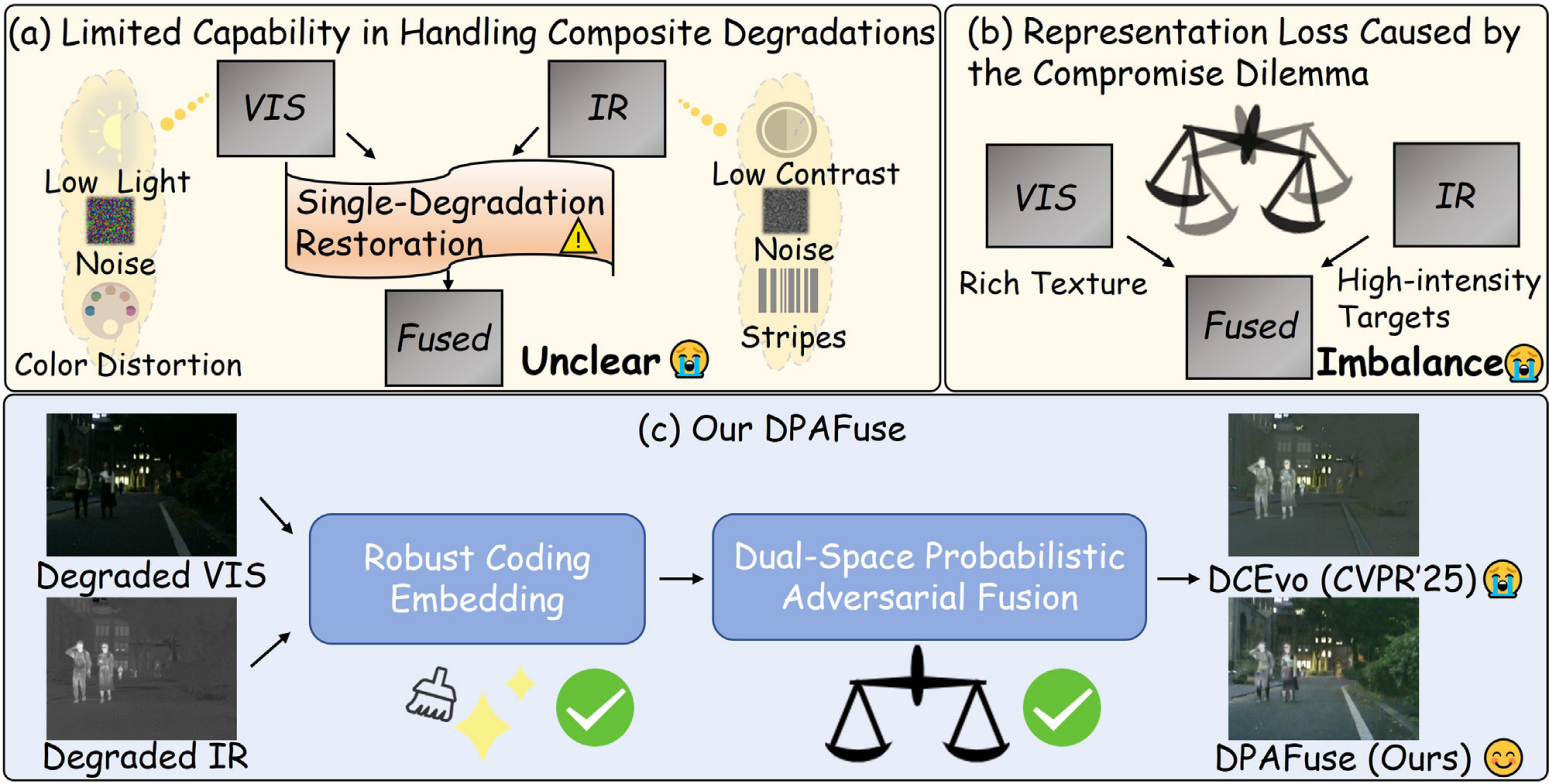
**Illustration of the motivation of this work.** (a) Limited capability in handling composite degradations; (b) representation loss caused by the compromise dilemma; (c) our proposed method DPAFuse.

Despite the progress made by the aforementioned methods, they still face two core challenges, as shown in Fig. 1. ***1) Limited capability in handling composite degradation.*** Although recent studies have introduced degradation-aware or degradation-removal techniques to improve fusion performance, most of them are designed to address only a single type of degradation. Even instruction-driven or text-guided methods typically process degradations sequentially, where each step targets a specific degradation. Such designs lack the capability to jointly model and suppress composite degradations, which are common in real-world imaging scenarios, such as low-light conditions accompanied by noise or sensor artifacts. As a result, the interaction and coupling effects among different degradations are often overlooked, leading to incomplete restoration and unreliable feature representations for subsequent fusion. Therefore, fusion networks require more robust and unified representation mechanisms that can simultaneously accommodate diverse and compound degradations. ***2) Representation loss caused by the compromise dilemma.*** Regardless of whether the method is traditional, deep non-end-to-end, or end-to-end, fusion strategies are prone to a “compromise dilemma”, where the network is forced to satisfy the conflicting requirements of resembling both visible and infrared images. Such pixel- or local-level compromises often lead to modal imbalance, weakening modality-specific representations and preventing the fused image from fully preserving complementary information. Addressing this issue requires a fusion mechanism capable of adaptive integration, rather than relying on simple averaging or selection rules.

To address the performance limitations of multi-modal image fusion under complex imaging conditions, this paper proposes a robust coding embedding-based probabilistic adversarial fusion framework called DPAFuse, as shown in Fig. 1 (c). The framework first constructs a unified latent feature space using a multi-modal autoencoder to extract structural and semantic information, with skip connections employed to preserve intermediate features. As a result, the distributional discrepancies among different composite degradations in the latent space are substantially reduced, which eases the difficulty of mapping degraded inputs to clean feature representations. Then, a robust coding embedding mechanism is applied to the encoded features, which suppresses degradation-related components and preserves stable modality-invariant structures, enabling the decoder to reconstruct clean and reliable fused representations. Based on this, a dual-space probabilistic adversarial fusion mechanism is introduced, where two discriminators guide the generator to produce fused images that simultaneously match the distributions in both feature and image spaces, and the structure-preserving loss further reinforces texture and edge details. Compared with conventional fusion strategies such as averaging or maximum selection, this method adaptively integrates multi-modal information at the global distribution level, improving the structural consistency, texture details, and target saliency of the fused images.

The main contributions of this work are as follows:

• We propose a robust coding embedding mechanism that enhances feature extraction under challenging conditions. Specifically, the autoencoder guides the aggregation of degradation-related information into the latent feature space, thereby compacting the feature distributions of composite degradations and bringing them closer to the underlying clean representation, while the robust coding embedding mechanism suppresses degradation components, yielding clean and modality-invariant structural and semantic features.
• We introduce a dual-space probabilistic adversarial fusion mechanism that enforces full-path distribution consistency. This mechanism enables the fusion network to align the distributions of fused results with source modalities at both the feature and image levels, and incorporates a structure-preserving loss to reinforce texture and edge preservation, ensuring that the fused images comprehensively inherit the complementary advantages of multi-modal inputs.
• Extensive experiments on public datasets demonstrate that DPAFuse delivers stronger recovery capability under composite degradation conditions and achieves superior fusion performance across all environments compared with existing approaches.

## 2 Related work

### 2.1 Degradation-aware image fusion methods

As deep learning techniques have been increasingly applied to infrared and visible image fusion, end-to-end frameworks such as U2Fusion [11] and CrossFuse [12] have demonstrated the effectiveness of unified feature learning and reconstruction under relatively ideal imaging conditions. However, in practical scenarios, fusion inputs are frequently affected by various degradations, motivating the incorporation of degradation-aware modules into fusion models. An early attempt in this direction is DIDFuse [10], which decomposes source images into background and detail components using a pre-trained autoencoder, aiming to alleviate the influence of noise and preserve structural details during fusion. Subsequent studies introduce explicit degradation modeling, particularly targeting illumination-related degradation. Representative methods such as DIVFusion [13] and DDBF [14] integrate degradation or illumination-aware priors into the fusion pipeline, allowing the model to adapt its fusion behavior under low-light conditions and improve visual quality in challenging lighting environments. These approaches demonstrate the benefit of incorporating degradation-specific cues into fusion frameworks. Beyond predefined degradation modeling, recent works explore instruction-driven fusion paradigms to enhance flexibility and controllability. Methods such as Text-IF [15] and InstructIVF [16] introduce textual or semantic instructions into the fusion process, enabling user-guided suppression of specific degradations and adaptive emphasis on different fusion objectives. By leveraging external guidance, these methods extend degradation awareness beyond fixed modeling strategies. Despite these advances, existing degradation-aware fusion methods generally address degradations in an isolated or prompt-wise manner, which limits their effectiveness in real-world scenarios where multiple degradations coexist. In contrast, our method aims to model composite degradation factors within a unified latent space, providing a more general and robust formulation for degradation-aware infrared and visible image fusion.

### 2.2 Generative adversarial networks

Generative Adversarial Networks (GANs) [17] were originally formulated as a minimax game for implicit distribution learning and have since evolved into a mature generative modeling paradigm through a series of methodological advances aimed at improving training stability and optimization behavior. Representative extensions include least-squares GAN (LSGAN) [18], which replaces the Jensen-Shannon divergence with a least-squares objective to alleviate vanishing gradients, and Wasserstein GAN with gradient penalty (WGAN-GP) [19], which enforces Lipschitz continuity to mitigate mode collapse. Conditional GANs (cGANs) [20] further enable controlled generation by introducing conditional inputs, while perceptual and feature-level constraints have been widely incorporated to enhance semantic fidelity in image generation tasks [21]. Benefiting from these developments, GANs have been extensively adopted for multimodal image fusion, where the fusion process can be naturally cast as an unsupervised generation problem. FusionGAN [22] represents an early attempt in this direction by constraining fused images to resemble the visible modality through adversarial learning while preserving infrared intensity information via the generator, and its subsequent extension [23] further improves thermal target representation by introducing gradient and edge-aware losses. To alleviate modality bias induced by single-modality supervision, dual-discriminator frameworks were proposed. Models like DDcGAN [24] and DUGAN [25] employ multi-discriminator strategies to align fused results with both infrared and visible distributions. Recent advancements further integrate high-level insights into the adversarial paradigm. For instance, SDSFusion [26] introduces semantic awareness for degraded scenes, while FreqGAN [27] leverages frequency-domain analysis to refine textural details. Despite their effectiveness, existing GAN-based fusion methods predominantly perform adversarial learning in the image domain, lacking explicit regulation of modality-specific feature distributions in latent space, which limits their ability to disentangle complementary thermal saliency and fine-grained visible textures. In contrast, our method introduces a dual-space probabilistic adversarial fusion framework that enables modality-aware discrimination across both latent and image domains, leading to more robust and balanced fusion.

## 3 Method

### 3.1 Problem formulation

**Fig. 2:**
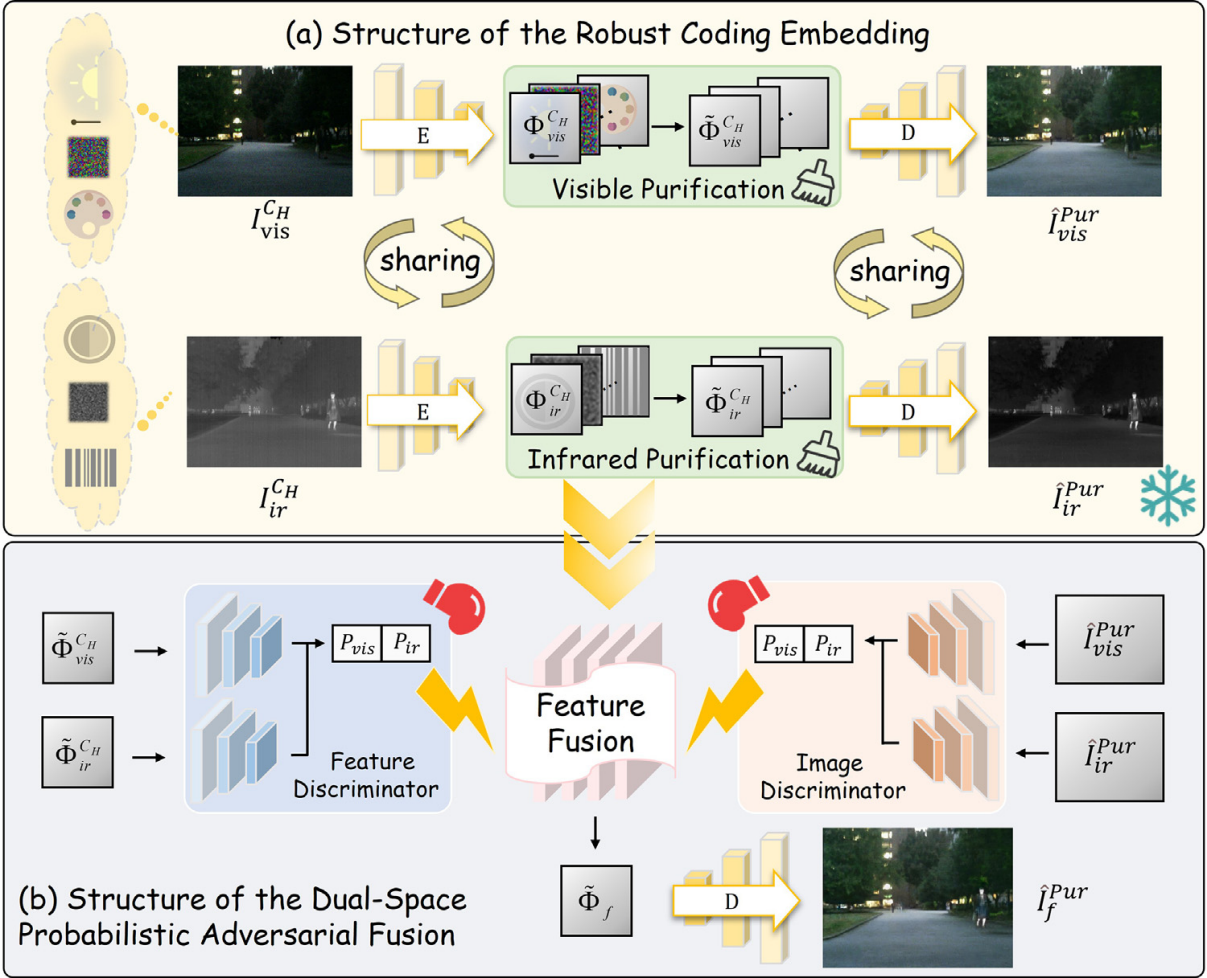
**Overall framework of our DPAFuse.** (a) Structure of the robust coding embedding; (b) structure of the dual-space probabilistic adversarial fusion.

As discussed in the Introduction, existing fusion methods are fundamentally challenged by two critical issues: limited capability in handling composite degradations and representation loss caused by the compromise dilemma. To effectively address these aforementioned challenges, this paper reformulates multimodal image fusion as a unified problem of robust information encoding and fusion optimization, as shown in Fig. 2. Let the infrared and visible images acquired under a compound degradation environment be denoted as $I_{ir}^{C_{H}}$ and $I_{vis}^{C_{H}}$, respectively, where $C_{H}$ represents the types of compound degradation present in the images. Specifically, in visible images, $C_{H}$ includes low illumination, noise, and color distortion, whereas in infrared images, it corresponds to low contrast, noise, and non-uniform striping artifacts. These types of noise generally cover the common degradation patterns observed in existing infrared and visible image fusion datasets.

To construct a stable and degradation-aware latent representation, we develop a Robust Coding Embedding (RCE) mechanism, which consists of two key components: a multimodal encoder–decoder architecture and a robust embedding module. The whole process can be expressed as:

(1) $${\hat{I}}_{M}^{\text{pur}}=RCE\left(I_{M}^{C_{H}}\right),$$

where RCE(·) denotes the proposed Robust Coding Embedding mechanism composed of the encoder-decoder backbone and the robust embedding refinement unit. Given an input $I_{M}^{C_{H}}$, the encoder E(·) projects degraded multimodal observations into a unified latent space, thereby contracting the distributional discrepancy among composite degradations and reducing their distance to the underlying clean representation, while preserving structural consistency and modality-specific information integrity. Building on this encoded representation, the robust embedding module further suppresses degradation-related components while preserving stable, modality-invariant structures. This refinement yields a more stable and consistent latent representation ${\tilde{\Phi }}_{M}^{C_{H}}$. Finally, the decoder reconstructs purified modality-specific outputs ${\hat{I}}_{M}^{\text{pur}}$ which can be leveraged in subsequent fusion tasks.

Building upon this, we address the second challenge of representation loss in the fusion process by designing a dual-space probabilistic adversarial fusion mechanism, which encourages the fused output to retain complementary information from both modalities. This framework consists of a fusion generator F and a pair of discriminators $\left\{D_{\text{Fea}},D_{\text{Img}}\right\}$. The generator F adaptively fuses the purified latent features to produce a fused representation ${\tilde{\Phi }}_{f}$ that maximizes information content. The feature discriminator DFea is trained to distinguish between the purified latent features of the visible and infrared modalities, ${\tilde{\Phi }}_{vis}^{C_{H}}$ and ${\tilde{\Phi }}_{ir}^{C_{H}}$, thereby aligning the latent distributions. Similarly, the image discriminator $D_{\text{Img}}$ distinguishes the reconstructed modality-specific images, ${\hat{I}}_{vis}^{\text{Pur}}$ and ${\hat{I}}_{ir}^{\text{Pur}}$, ensuring that the fused image preserves high-quality modality characteristics while remaining indistinguishable from the source reconstructions. The overall optimization is formulated as a min-max game:

(2) $$\min_{F}\max_{D_{\text{Fea}},D_{\text{Img}}}V\left(F,D_{\text{Fea}},D_{\text{Img}}\right),$$

where V measures the discriminators’ ability to separate the fused outputs from the source modalities. By jointly optimizing this adversarial objective together with structural fidelity constraints, the framework encourages the fusion generator to produce high-quality, cross-modal aligned, and information-preserving fused images, completing the end-to-end process from degraded inputs $I_{M}^{C_{H}}$ to refined fusion outputs.

### 3.2 Robust coding embedding

#### 3.2.1 Latent space construction

To achieve robust multimodal image fusion under composite degradation, we follow the mechanism proposed in OmniFuse [28] to construct a high-quality latent space. Our multimodal encoder-decoder network maps degraded source images into a unified latent representation, providing clean and structurally complete features for subsequent fusion.

Specifically, the input multimodal degraded image $I_{M}^{C_{H}}$ (where M denotes the modality type and $C_{H}$ represents the composite degradation) is first mapped into latent features by the encoder. Meanwhile, to better preserve the structural information of the scene, a skip-connection mechanism is introduced to synchronously extract multi-level intermediate features during encoding. The overall encoding process can be formulated as:

(3) $$\begin{array}{c} \left\{\Phi_{M}^{C_{H}},\Omega_{M}^{C_{H}}\right\}=E\left(I_{M}^{C_{H}}\right), \end{array}$$

where $\Phi_{M}^{C_{H}}$ denotes the compressed latent features, which encode high-dimensional semantic information as well as degradation noise, and $\Omega_{M}^{C_{H}}$ records structural information from multiple levels to assist in the fine reconstruction of the image. During decoding, the latent and intermediate features are jointly fed into the decoder to reconstruct the original image space:

(4) $$\begin{array}{c} {\hat{I}}_{M}^{C_{H}}=D\left(\Phi_{M}^{C_{H}},\Omega_{M}^{C_{H}}\right). \end{array}$$

**Fig. 3:**
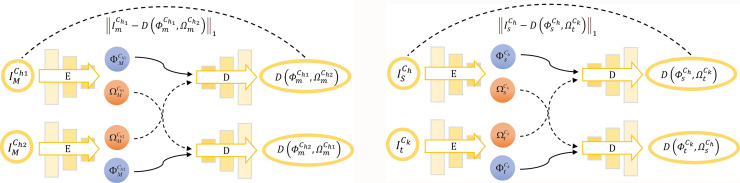
**Illustration of the cross-modal feature reorganization mechanism.** (a) Intra-modal degradation reorganization constraint; (b) cross-modal reorganization constraint.

To effectively compress degradation information into the latent features while preserving structural information within the middle features, we design a cross-modal feature reorganization mechanism, illustrated in Fig. 3, to guide the training of the encoder-decoder network in a more structured and robust manner. This mechanism consists of two parts:

1) Intra-modal degradation reorganization constraint $L_{\mathrm{intra}}$. To further regulate the distribution of degradation-related information, this constraint targets images from the same modality but with different degradation types. By reorganizing their middle features, the reconstructed results should remain unchanged, indicating that the degradation properties are primarily determined by the latent features. The corresponding loss is defined as:

(5) $$\begin{array}{c} L_{\mathrm{intra}}=\sum_{m}\sum_{h_{1}}\sum_{h_{2}}{∥I_{m}^{C_{h1}}-D\;\left(\Phi_{m}^{C_{h1}},\Omega_{m}^{C_{h2}}\right)∥}_{1}. \end{array}$$

2) Cross-modal reorganization constraint $L_{\mathrm{cross}}$. For images from different modalities but depicting the same scene, their middle features are expected to maintain consistency. Specifically, the middle features extracted from different modalities should be mutually interchangeable and complementary in representing the scene information. The corresponding loss is defined as:

(6) $$\begin{array}{c} L_{\mathrm{cross}}=\sum_{\begin{array}{c} s,t\\ s\neq t \end{array}}\sum_{h}\sum_{k}{∥I_{s}^{C_{h}}-D\;\left(\Phi_{s}^{C_{h}},\Omega_{t}^{C_{k}}\right)∥}_{1}. \end{array}$$

These two reorganization constraints collaboratively guide the latent features to encode degradation-related information, while the middle features capture modality-invariant structural information, thereby achieving functional disentanglement. The overall training objective of the autoencoder is formulated as follows: $L_{\text{AE}}=L_{\text{intra}}+L_{\text{cross}}$. By optimizing this objective function, the latent space acquires representation capability suitable for modeling and restoring degradations, thereby providing a robust foundation for subsequent information fusion based on adversarial or diffusion mechanisms.

#### 3.2.2 Robust embedding

**Fig. 4:**
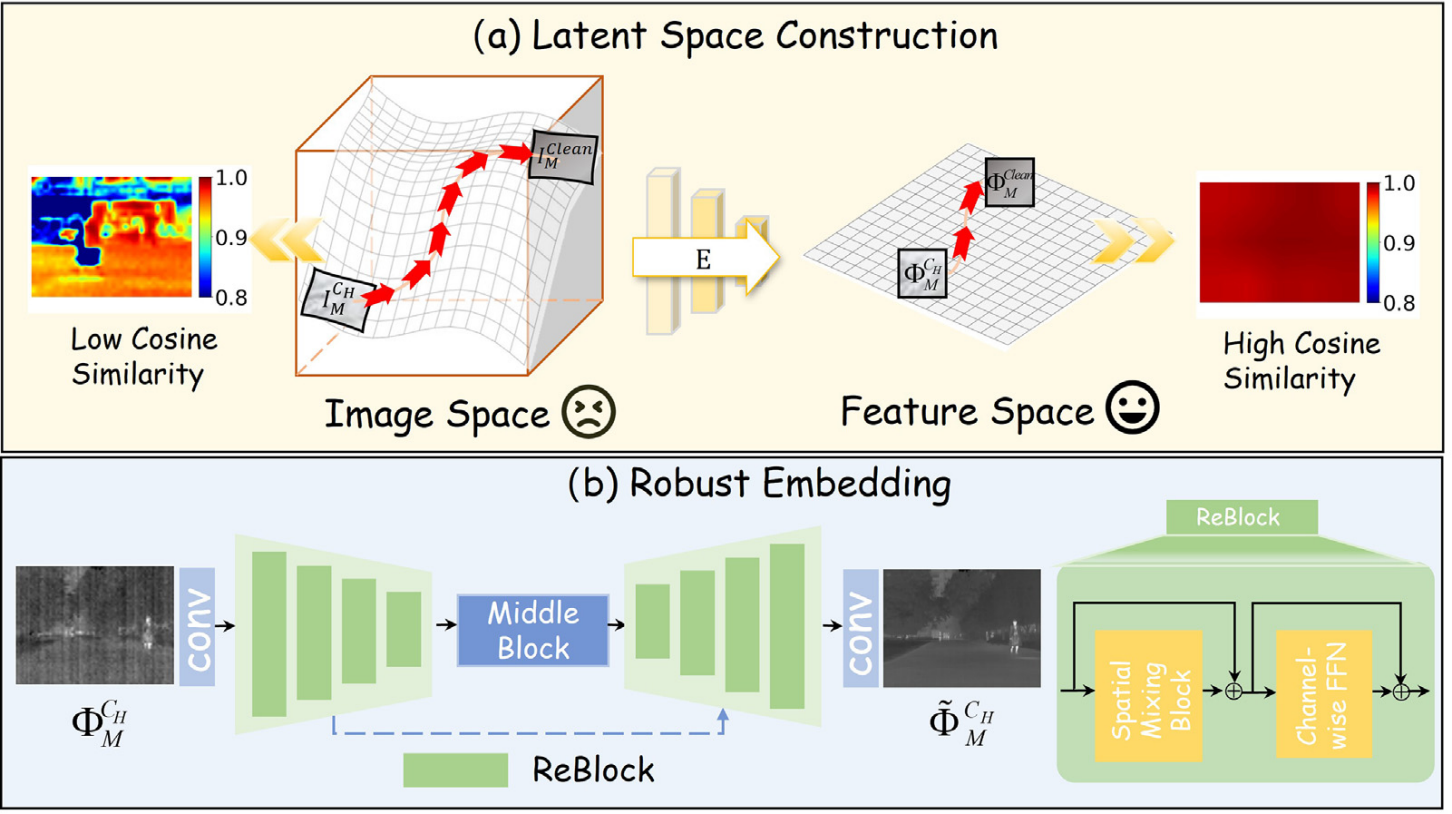
**Illustration of the robust coding embedding mechanism.** (a) Latent space construction; (b) robust embedding module.

To further enhance the stability and discriminability of the latent space under composite degradation conditions, we incorporate a Robust Embedding module on top of the initial latent representation. The module operates within the latent feature space, where representations directly encode high-level semantic information and form a more compact and structured manifold. Such a latent embedding not only shortens feature-space distances compared with the image domain, but also contracts the distributional discrepancies among diverse composite degradations and aligns them more closely with the underlying clean representation, thereby facilitating more effective modeling and suppression of composite degradations while preserving critical semantic and structural information, as illustrated in Fig. 4.

Specifically, we design a nonlinear mapping module that takes the raw latent features obtained from the multimodal encoder as input and learns a purified latent representation:

(7) $$\begin{array}{c} {\tilde{\Phi }}_{M}^{C_{H}}=RE\left(\Phi_{M}^{C_{H}}\right), \end{array}$$

where RE(·) denotes a learnable mapping function composed of residual units and attention mechanisms. Within the encoder-decoder architecture, this module leverages residual blocks and attention mechanisms to enhance feature extraction. At each encoder stage, the RE modules progressively extract semantic representations through residual blocks, attention mechanisms, and nonlinear gating, while downsampling operations expand the receptive field to capture global structural information. In the middle blocks, residual and attention mechanisms further strengthen the modeling of degradation patterns. During decoding, upsampling restores spatial resolution, and skip connections incorporate high-resolution features, enabling the network to focus on structural boundaries and critical semantic regions while effectively suppressing redundant or degraded information. As a result, the output latent features are more concentrated, stable, and semantically meaningful.

**Fig. 5:**
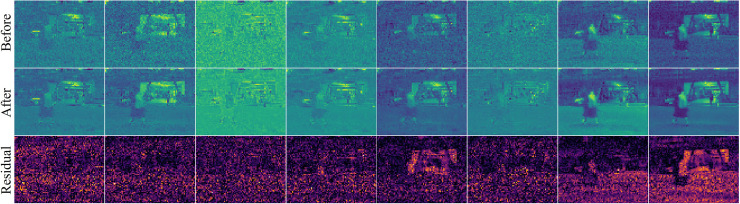
**Channel-wise latent responses before and after robust embedding module**.

To further illustrate the effect of the Robust Embedding module, we visualize the channel-wise latent features before and after RE, together with their residual maps, as shown in Fig. 5. The purified features exhibit more concentrated and structurally coherent activations compared to the scattered, degradation-sensitive responses in the raw latent space. The residual maps primarily highlight degradation-corrupted regions rather than semantic boundaries, indicating that RE effectively suppresses degradation components while preserving intrinsic structural information.

During training, to guide this module in effectively compressing degradation information while preserving semantic content, we introduce a dual consistency constraint at both the feature and image levels.

First, at the feature level, we require that the purified latent features be as close as possible to the “clean” latent representations obtained from degradation-free inputs of the same modality. Accordingly, the feature-level consistency loss is defined as:

(8) $$\begin{array}{cc} L_{\text{fea}} & ={∥{\tilde{\Phi }}_{M}^{C_{H}}-\Phi_{M}^{\text{clean}}∥}_{1}. \end{array}$$

This constraint forces the network to converge toward the semantic center of the clean data, thereby progressively compressing the degradation components within the latent features.

Second, at the image level, the purified latent features $\Omega_{M}^{C_{H}}$ are fed into the decoder D together with the original middle features to reconstruct the image ${\hat{I}}_{M}^{\text{pur}}$.The reconstructed image is then compared with the degradation-free reference $I_{M}^{\text{clean}}$, and the reconstruction loss is defined as follows:

(9) $$\begin{array}{cc} L_{\text{image}} & ={∥{\hat{I}}_{M}^{\text{pur}}-I_{M}^{\text{clean}}∥}_{1}. \end{array}$$

This supervision ensures that the purified latent features effectively suppress degradation-related noise while preserving complete semantic structures and reconstruction capability, thereby preventing the loss of critical regions during degradation compression.

Finally, the optimization objective of the Robust Embedding module is formulated as: $L_{\text{RE}}=L_{\text{fea}}+L_{\text{image}}.$

### 3.3 Dual-space probabilistic adversarial fusion

**Fig. 6:**
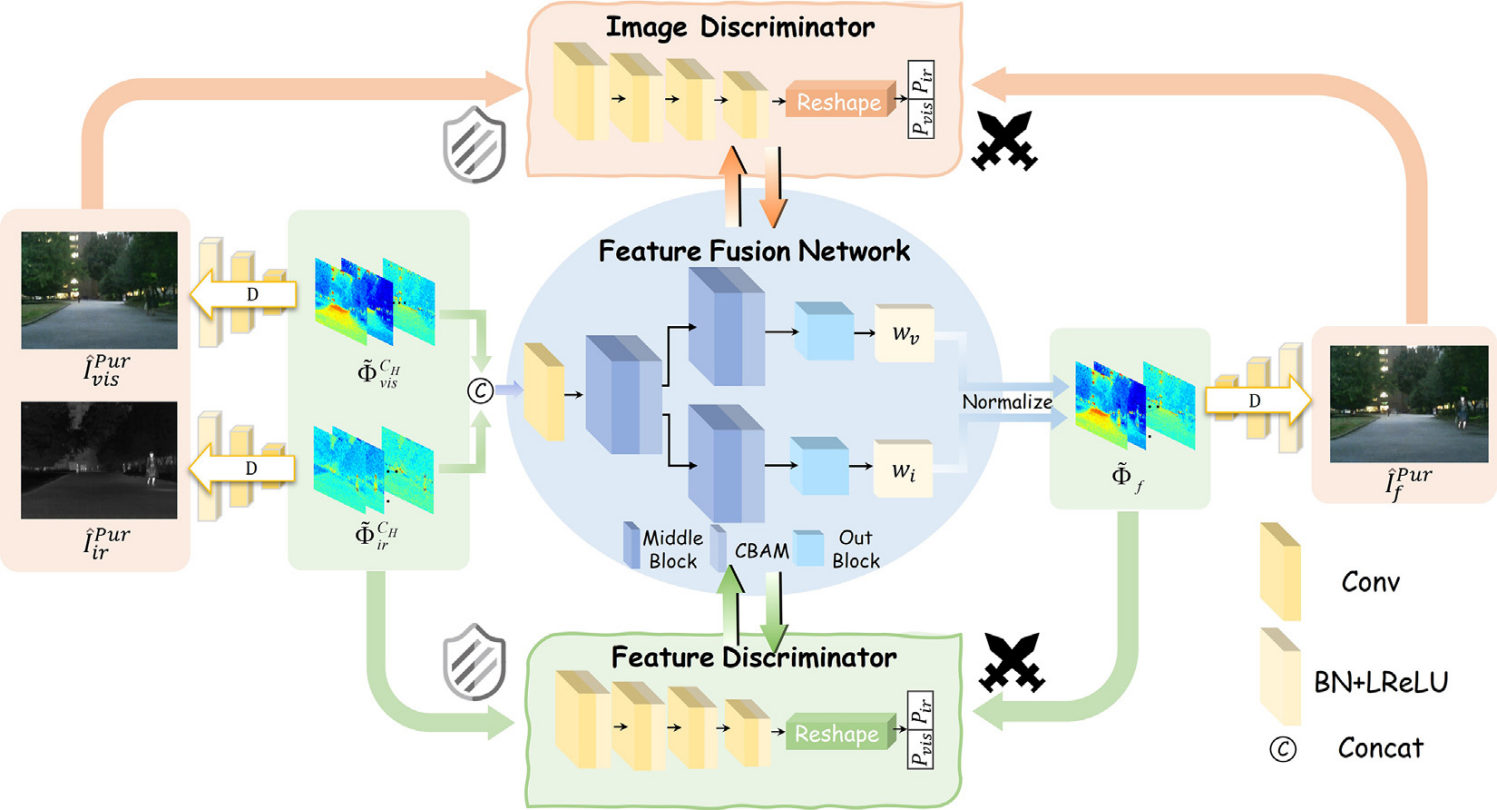
**Network architecture for the dual-space probabilistic adversarial fusion**.

After obtaining purified and information-rich latent features ${\tilde{\Phi }}_{M}^{C_{H}}$ through the robust coding embedding module, the key challenge lies in how to optimally fuse these high-quality representations. Accordingly, we propose a novel adaptive fusion paradigm jointly driven by adversarial learning and structure preservation. The essence of this method lies in constructing a dynamic and learnable fusion process that achieves source modality alignment at the distribution level through adversarial training, while an explicit constraint is imposed to preserve critical structural details of the images. The framework consists of a fusion network serving as the generator and two multi-class discriminators a feature discriminator $D_{Fea}$ and an image discriminator $D_{Img}$, as shown in Fig. 6.

For the generator, we design a fusion network F that takes the purified latent features from the two modalities ${\tilde{\Phi }}_{ir}^{C_{H}}$ and ${\tilde{\Phi }}_{vis}^{C_{H}}$ as input and adaptively produces the fused latent feature ${\tilde{\Phi }}_{f}$. Specifically, the generator first concatenates the two input latent features and passes them through an initial convolutional block for feature integration. The resulting feature stream is then fed into a core processing unit composed of multiple cascaded channel-spatial attention blocks, enabling the network to dynamically focus on the most informative channels and spatial regions within the feature maps. Subsequently, a dual-branch structure is employed, where each branch independently processes the attention-weighted features and ultimately outputs a spatial weight map through convolutional layers. The two spatial weight maps are denoted as $w_{i}$ and $w_{v}$, representing the respective fusion contributions of the two input modalities. The final fused latent representation ${\tilde{\Phi }}_{f}$ is then obtained by performing a pixel-wise adaptive weighted summation of the input latent features, formulated as:

(10) $$\begin{array}{cc} {\tilde{\Phi }}_{f} & =\frac{w_{i}}{w_{i}+w_{v}}⊙{\tilde{\Phi }}_{ir}^{C_{H}}+\frac{w_{v}}{w_{i}+w_{v}}⊙{\tilde{\Phi }}_{vis}^{C_{H}}, \end{array}$$

where ⊙ denotes element-wise multiplication.

In addition, for the intermediate features required by the decoder, we adopt a simple averaging fusion strategy:

(11) $$\begin{array}{cc} \Omega_{f} & =\frac{\Omega_{ir}^{C_{H}}+\Omega_{vis}^{C_{H}}}{2}. \end{array}$$

To effectively guide the generator, we design two structurally similar discriminators $D_{Fea}$ and $D_{Img}$ with different scopes. Although operating in different domains, both discriminators are formulated under a unified probabilistic objective, which enforces modality indistinguishability while preserving complementary information. This shared objective ensures that their supervision signals are inherently aligned rather than contradictory. Both are based on a convolutional sliding-window mechanism to enhance sensitivity to local textures and details, and they map their outputs to a two-dimensional probability vector via global pooling, thereby enforcing modality discrimination in the latent feature space and quality constraints in the image space, jointly improving the generator’s robustness and the reliability of the fusion results.

Specifically, the feature discriminator $D_{Fea}$ takes the latent feature maps as input and first performs preliminary feature extraction through a convolutional layer, followed by a LeakyReLU activation. It then stacks several convolution-normalization-activation blocks to progressively enhance the discriminative power of the feature representations. Finally, the output layer employs a 2D convolution to map the feature maps to a two-dimensional prediction vector, where the two components represent the probability of the input features belonging to the infrared features and the probability of belonging to the visible features, respectively.

Correspondingly, the image discriminator $D_{\text{Img}}$ takes the three-channel fused image as input. It adopts a structure similar to the feature discriminator but employs larger convolutional kernels and strides to better capture spatial consistency and structural integrity in cross-modal fusion. The output is a two-dimensional probability vector indicating whether the input image is closer to infrared or visible features.

We optimize the framework using a two-stage adversarial training strategy, forming a dynamic min-max game. In this process, the two discriminators constrain the generator at the feature and image levels, respectively, enabling multi-level adversarial optimization.

In the first stage, we optimize the two multi-class discriminators separately. For the feature discriminator $D_{Fea}$, its task is to distinguish between real purified latent features and the fused latent features generated by the generator. The loss function $L_{D}^{\text{Fea}}$ is defined as:

(12) $$\begin{array}{ccc} L_{D}^{\text{Fea}} & = & \alpha_{1}\;E\left[∥D_{\text{Fea}}\left({\tilde{\Phi }}_{ir}^{C_{H}}\right)-\left(P_{ir},P_{vis}\right){∥}_{2}^{2}\right]\\ & & +\alpha_{2}\;E\left[∥D_{\text{Fea}}\left({\tilde{\Phi }}_{vis}^{C_{H}}\right)-\left(P_{ir},P_{vis}\right){∥}_{2}^{2}\right]\\ & & +\alpha_{3}\;E\left[∥D_{\text{Fea}}\left({\tilde{\Phi }}_{f}\right)-\left(P_{ir},P_{vis}\right){∥}_{2}^{2}\right], \end{array}$$

where E denotes the expectation over all samples, computing the average loss across the entire data distribution; the parameters $\alpha_{1},\alpha_{2}$ and $\alpha_{3}$ are used to adjust the relative weights of the loss terms. The discriminator outputs a two-dimensional probability vector $\left(P_{ir},P_{vis}\right)$ for each input feature block. For the infrared features, we set the target probability to (1,0), indicating that the discriminator is expected to recognize the input as infrared rather than visible features. Similarly, for the visible features, the target probability is set to (0,1),indicating that the discriminator should recognize the input as visible rather than infrared features. For the fused features, the target probability is set to (0.5,0.5), which corresponds to the decision boundary of the binary discriminator. In this setting, the fused representation is explicitly encouraged to lie in a modality-indistinguishable region of the latent space, where the discriminator cannot confidently classify it as either infrared or visible. This probabilistic constraint can be interpreted as enforcing modality neutrality or modality balance. Rather than performing simple feature averaging, the objective promotes distribution-level indistinguishability under adversarial supervision, thereby preventing the generator from collapsing toward a dominant modality. As a result, shared structural and semantic information is preserved while modality-specific biases are suppressed.

For the image discriminator, its task is to distinguish between the “real” images reconstructed from the purified latent features and the forged fused images. Similarly, the loss function $L_{D}^{\text{Img}}$ is defined as:

(13) $$\begin{array}{ccc} L_{D}^{\text{Img}} & = & \alpha_{1}\;E\left[∥D_{\text{Img}}\left({\hat{I}}_{ir}^{\text{Pur}}\right)-\left(P_{ir},P_{vis}\right){∥}_{2}^{2}\right]\\ & & +\alpha_{2}\;E\left[∥D_{\text{Img}}\left({\hat{I}}_{vis}^{\text{Pur}}\right)-\left(P_{ir},P_{vis}\right){∥}_{2}^{2}\right]\\ & & +\alpha_{3}\;E\left[∥D_{\text{Img}}\left({\hat{I}}_{f}^{\text{Pur}}\right)-\left(P_{ir},P_{vis}\right){∥}_{2}^{2}\right]. \end{array}$$

In the second stage, we fix the parameters of the discriminators and optimize the generator $G_{Fuse}$ instead.The generator aims to produce fused results that can ”fool” both discriminators while maximally preserving the structural details of the source images. Accordingly, the generator’s loss function $L_{G}^{\text{Fuse}}$ consists of two components: the adversarial loss $L_{G}^{\text{adv}}$ and the structure-preserving loss $L_{\text{struct}}$: $L_{G}^{\text{Fuse}}=L_{G}^{\text{adv}}+L_{\text{struct}}.$ Here, the adversarial loss $L_{G}^{\text{adv}}$ is designed to encourage the generator’s outputs, whether features or images, to exhibit both infrared and visible characteristics from the perspective of the discriminators, ensuring that their predicted probabilities approach a balanced distribution (0.5,0.5). In other words, the generator is adaptively optimized to make it difficult for the discriminators to clearly classify the fused results as either infrared or visible, thereby effectively enhancing modality indistinguishability. Importantly, both adversarial losses push the fused outputs toward the same probabilistic target (0.5,0.5) and update a shared weight-generation pathway in the generator. As a result, the gradients propagated from $D_{Fea}$ and $D_{Img}$ are naturally coordinated in direction, preventing adversarial competition across representation spaces. The generator is formally expressed as:

(14) $$\begin{array}{ccc} L_{G}^{\text{adv}} & = & \lambda_{\text{fea}}\;E\left[∥D_{\text{Fea}}\left({\tilde{\Phi }}_{f}\right)-\left(P_{ir},P_{vis}\right){∥}_{2}^{2}\right]\\ & & +\lambda_{\text{img}}\;E\left[∥D_{\text{Img}}\left({\hat{I}}_{f}^{\text{Pur}}\right)-\left(P_{ir},P_{vis}\right){∥}_{2}^{2}\right]. \end{array}$$

The structure-preserving loss $L_{\text{struct}}$ directly operates on the image content. By comparing the gradient information of the luminance channel, it encourages the fused image to inherit the strongest edges and textures from both source images, thereby preserving more structural details. Specifically, the loss is defined as:

(15) $$\begin{array}{c} L_{\text{struct}}=∥\nabla Y\left({\hat{I}}_{f}^{\text{Pur}}\right)-\max(\nabla Y\left({\hat{I}}_{ir}^{\text{Pur}}\right),\nabla Y\left({\hat{I}}_{vis}^{\text{Pur}}\right)){∥}_{2}^{2}, \end{array}$$

where ∇Y denotes the gradient magnitude computed on the luminance channel in the YCbCr color space, using the sobel operator. Importantly, this loss focuses solely on local structural consistency by taking the maximum gradient at each pixel, and thus does not involve global averaging or cross-modal compromise; it only preserves the most salient structures from each modality without affecting the overall modal balance.

By jointly optimizing the adversarial and structure-preserving losses, our model can generate fused results that are both highly realistic and modality-ambiguous, while accurately preserving complementary information from the multiple source modalities, thereby ultimately achieving robust, reliable, and high-fidelity fusion results.

## 4 Experiments and results

### 4.1 Implementation details

#### 4.1.1 Datasets

To cover a wide range of typical degradation types, we construct the required training data based on several public datasets, including MFNet [29], FMB [30], LLVIP [31], and RoadScene [11]. Specifically, during the training stage of the robust coding embedding mechanism, 1800 visible and 1800 infrared images are selected from the MFNet, LLVIP, FMB, and RoadScene datasets. These samples cover four representative scenarios, namely cross-modal, infrared, visible, and non-degraded cases, providing a comprehensive representation of diverse imaging conditions. To obtain “pseudo-clean” reference images that can serve as supervision signals, existing image restoration methods (such as QuadPrior [32] and Diff-Retinex [33]) are employed to denoise, enhance, and correct degraded images. The best results are then manually selected to approximate real clean images as closely as possible, thereby avoiding the limitations of relying on a single restoration algorithm. For the dual-space probabilistic adversarial fusion mechanism training, 920 images from the MFNet, FMB, and LLVIP datasets are used to train the network to learn how to effectively combine complementary information from visible and infrared modalities, ensuring that the fused outputs retain critical details from both sources while maintaining structural and semantic consistency.

#### 4.1.2 Training details

In terms of the training process, the autoencoder is first trained to establish a suitable latent space representation. Subsequently, the robust autoencoders are trained separately on the infrared and visible modalities, enabling the model to stably extract and represent modality-specific features under various degradation conditions. Finally, the weights of the first two parts are frozen, and the fusion module is trained to achieve cross-modal feature alignment and reorganization, thereby generating fused results that exhibit superior structural consistency and information integrity. The detailed training procedure of the fusion module is summarized in Algorithm 1.

**Table:** **Algorithm 1 Training Procedure of Dual-Space Adversarial Fusion**.

For the dual-space probabilistic adversarial fusion, both the feature-level adversarial loss $L_{D}^{\text{Fea}}$ and the image-level adversarial loss $L_{D}^{\text{Img}}$ consist of three weighted components. The corresponding coefficients are set to $\alpha_{1}=0.25$, $\alpha_{2}=0.25$, and $\alpha_{3}=0.5$ in all experiments. For the optimization setup, the robust autoencoder is trained using the Lion optimizer with an initial learning rate of 5e-6, combined with a cosine annealing learning rate schedule. The autoencoder and fusion module are also trained with the Lion optimizer, using an initial learning rate of 3e-5, while keeping the other configurations consistent with those of the robust autoencoder. All experiments are conducted on a server equipped with four NVIDIA GeForce RTX 3090 GPUs (each with 24 GB of VRAM) and an AMD EPYC 7H12 64-core CPU.

#### 4.1.3 Evaluation strategy

We compare DPAFuse with nine advanced fusion approaches, including CrossFuse [12], DCEvo [34], Diff-IF [35], LRRNet [36], MaeFuse [37], SHIP [38], DDBF [14], DAFusion [39], and Text-IF [15]. These methods represent the leading techniques in the field of image fusion, among which Text-IF supports dynamic control of the fusion process by allowing text-based instructions to specify the degradation type in the source images. In our experimental setup, the visible images suffer from low illumination, noise, and color distortion, while the infrared images exhibit low contrast, noise interference, and noticeable stripe artifacts. To ensure a fair comparison, all competing methods were preprocessed before fusion. Specifically, the visible images were enhanced using Instruct-IR [40] with two sequential instructions for denoising and low-light enhancement. For infrared images, ASCNet [41] was employed to remove stripe artifacts, Restormer [42] was applied for denoising, and a dark channel prior-based method [43] was used for infrared enhancement. Finally, the fusion results were evaluated using four metrics: MI [44], Qabf [45], PSNR [46], and SD [47], to comprehensively assess the performance in terms of information preservation, structural consistency, and visual quality.

### 4.2 Comparative experiments on MFNet dataset

#### 4.2.1 Qualitative analysis

**Fig. 7:**
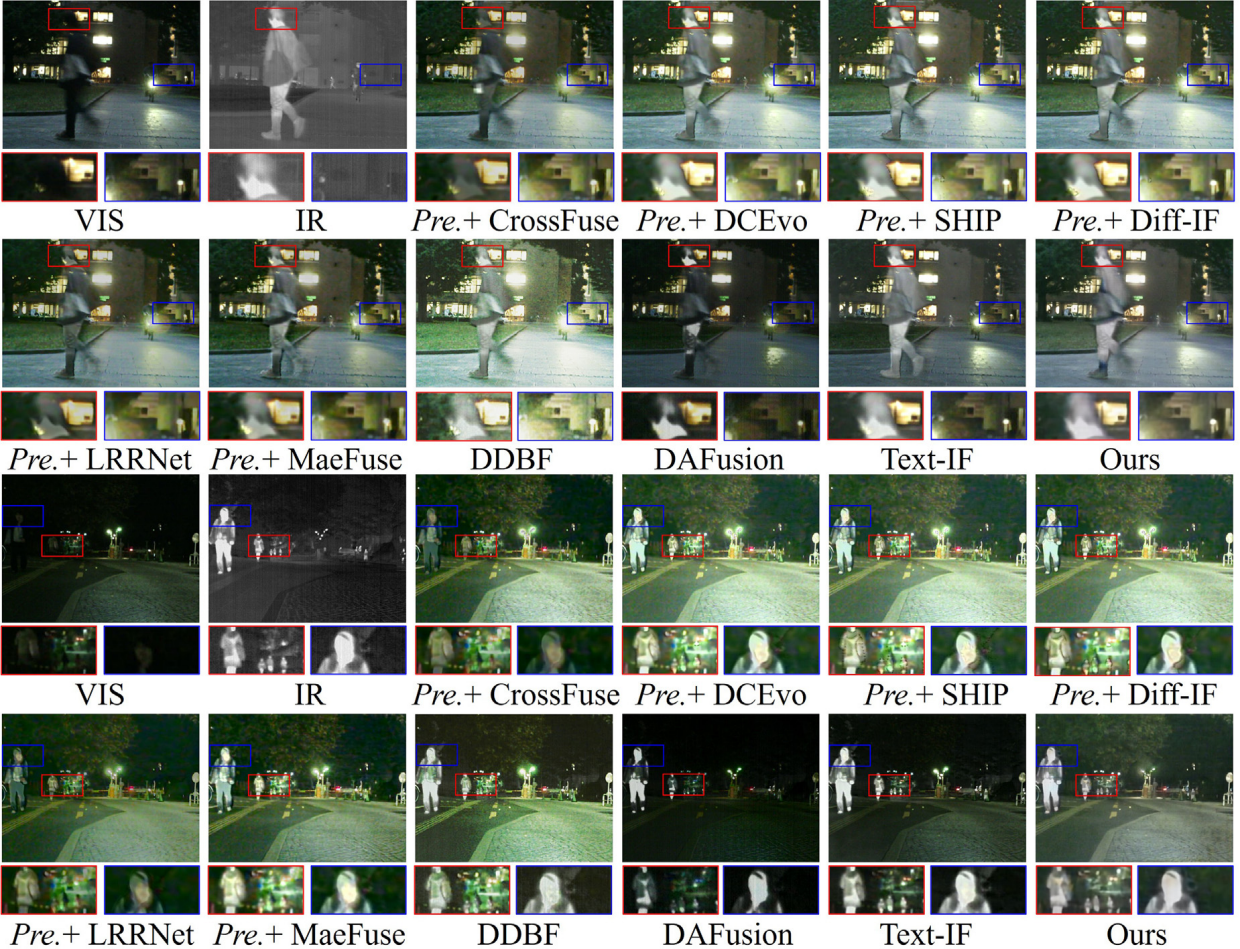
**Qualitative results of the comparative experiment on the MFNet dataset.***Pre.* indicates fusion results obtained from images preprocessed using external restoration and enhancement methods.

We conducted comparative experiments on the MFNet dataset to evaluate DPAFuse under composite degradation conditions, where both infrared and visible images are simultaneously affected by multiple degradations, including low-light illumination, noise contamination, stripe artifacts, and color distortions. Fig. 7 presents the qualitative results. Overall, DPAFuse demonstrates clear advantages: it preserves salient infrared targets and visible-light texture details, enhances brightness under low-light conditions, and corrects color distortions, producing natural and complete fused images.

The compared advanced methods can be categorized into two groups for in-depth analysis. The first group comprises image-level pre-processing approaches. Experimental results indicate that such cascaded pipelines often fail to fully recover severely degraded regions. Although the aforementioned pre-processing workflow described in Section 4.1.3 can improve input quality to some extent, a task mismatch frequently persists between the restored images and the subsequent fusion model under composite degradation conditions. This often results in insufficient brightness or blurred object boundaries, while the thermal saliency of distant pedestrians is weakened even though background textures are relatively well preserved. Furthermore, the lack of consistency constraints between restoration and fusion stages tends to introduce artificial artifacts or unnatural color tones in the final outputs. The second group consists of fusion methods with inherent degradation-removal capabilities. While these methods attempt to handle degradations internally, they often struggle to jointly suppress multiple degradation factors. Thus, although thermal targets are usually highlighted with clear edges, visible-light details such as road structures or vegetation textures are partially lost, causing the fused images to resemble enhanced infrared outputs. Moreover, residual low-light effects and color distortions are frequently observed.

In contrast, DPAFuse deeply couples degradation suppression with information fusion at the feature level. This joint design enables the model to adaptively address composite degradation challenges. As a result, the generated images outperform both groups in terms of brightness consistency and color fidelity, achieving an optimal balance between highlighting thermal saliency and preserving environmental textures.

#### 4.2.2 Quantitative analysis

A total of 100 images were selected from the MFNet dataset for quantitative analysis. The comparison results are shown in Table 1, where the bolded values indicate the best performance and the underlined values indicate the second-best. It can be observed that DPAFuse achieves the highest scores in MI, Qabf, and PSNR, while ranking fourth in SD. This indicates that the proposed method offers significant advantages in multi-modal information complementarity and structural preservation, while effectively enhancing the fidelity of reconstructed images. Although the SD score is slightly lower than that of some compared methods, this difference reflects the trade-off between detail enhancement and noise suppression. Overall, DPAFuse can generate fused results that maintain image details and global consistency while achieving superior visual coherence and information integrity.

**Table 1:** **Quantitative results on the MFNet dataset**.

### 4.3 Comparative experiments on LLVIP dataset

#### 4.3.1 Qualitative analysis

**Fig. 8:**
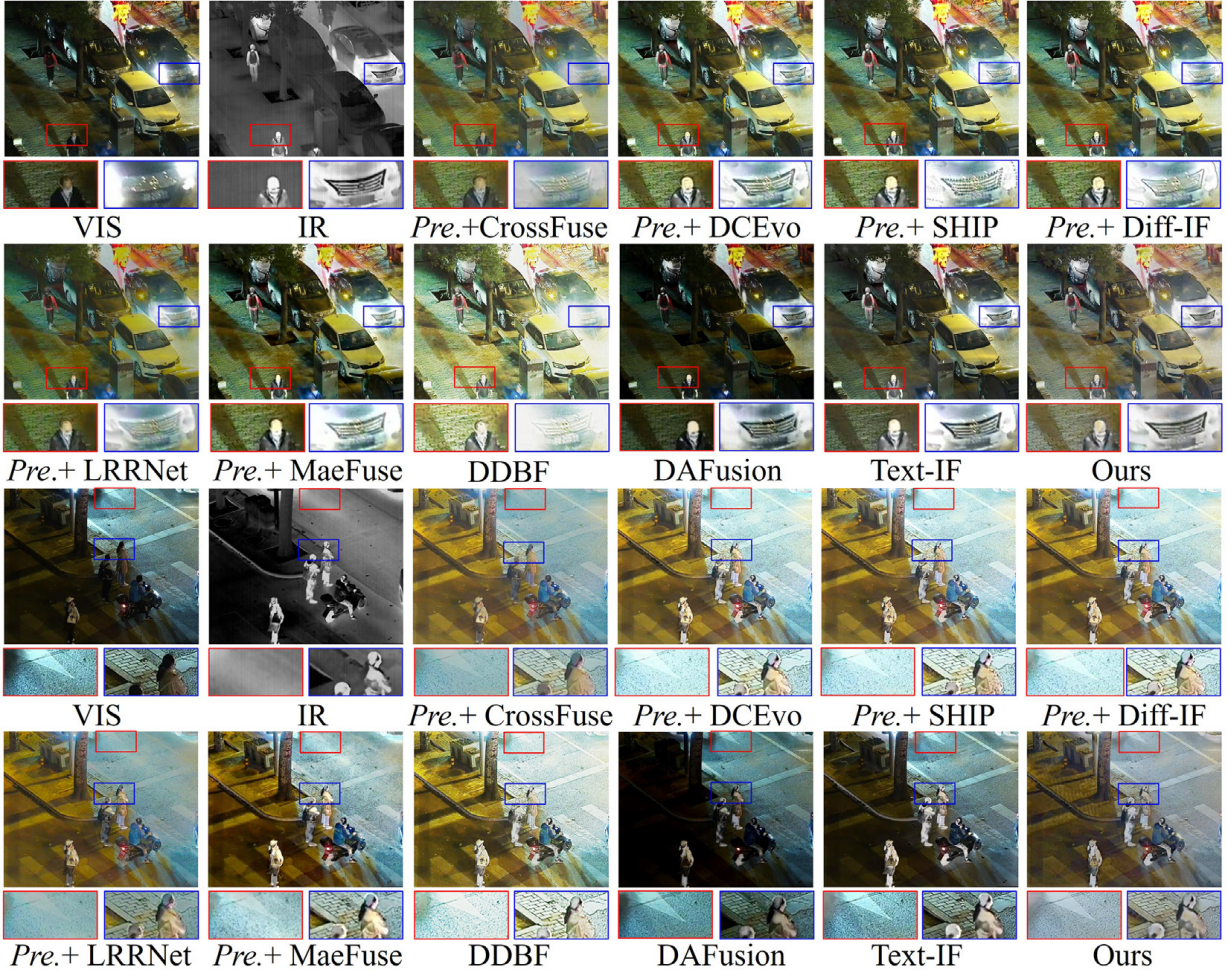
**Qualitative results of the comparative experiment on the LLVIP dataset.***Pre.* indicates fusion results obtained from images preprocessed using external restoration and enhancement methods.

We further evaluate DPAFuse on the LLVIP dataset to assess its robustness in real-world low-light scenarios, with qualitative comparisons illustrated in Fig. 8. In addition to severe low illumination, the images also exhibit composite degradations, including noise interference, subtle stripe artifacts, and color distortions. Methods that rely on explicit image-level enhancement prior to fusion tend to emphasize global illumination improvement, yet often exhibit limited capability in preserving fine structural details under low-light conditions. As shown in the first group of Fig. 8, such approaches frequently fail to maintain a clear distinction between foreground pedestrians and surrounding background objects. Although pedestrian brightness is partially improved, background vehicles and road textures are either over-smoothed or insufficiently recovered, resulting in a loss of scene completeness. Another class of methods incorporates degradation handling within the fusion network itself. These approaches generally succeed in highlighting thermal targets, particularly pedestrian contours, but often do so at the expense of visible-light details. In the second group of Fig. 8, this imbalance is manifested by weakened road surface textures and suppressed structural information, causing the fused images to appear dominated by infrared responses rather than presenting a coherent multimodal representation.

By comparison, DPAFuse achieves a more balanced fusion outcome in low-light environments. Foreground pedestrians are rendered with clear contours and enhanced thermal saliency, while background elements such as vehicles and road textures remain well preserved. Notably, the grille details of background vehicles and the fine surface patterns of the road are distinctly retained without introducing excessive brightness or darkness. This demonstrates that DPAFuse effectively harmonizes infrared prominence with visible contextual information, leading to a more faithful and visually coherent scene representation.

#### 4.3.2 Quantitative analysis

A total of 100 image pairs from the LLVIP dataset were selected for quantitative evaluation, and the comparison results are presented in Table 2. It can be observed that DPAFuse achieves the highest performance in both MI and PSNR metrics, demonstrating its strong capability in capturing multimodal complementarity and structural preservation. Meanwhile, it also maintains competitive results across other metrics, indicating a well-balanced performance overall. These findings suggest that DPAFuse effectively integrates information from multiple sources while further enhancing visual consistency and information completeness.

**Table 2:** **Quantitative results on the LLVIP dataset**.

### 4.4 Robustness evaluation

#### 4.4.1 Asymmetric degradation scenarios

In practical applications, it is common for only one modality to suffer from severe degradation while the other remains relatively clean. To evaluate the robustness of our framework under such asymmetric conditions, we conducted extensive experiments where degradation was applied to either the visible or infrared modality independently.

**Fig. 9:**
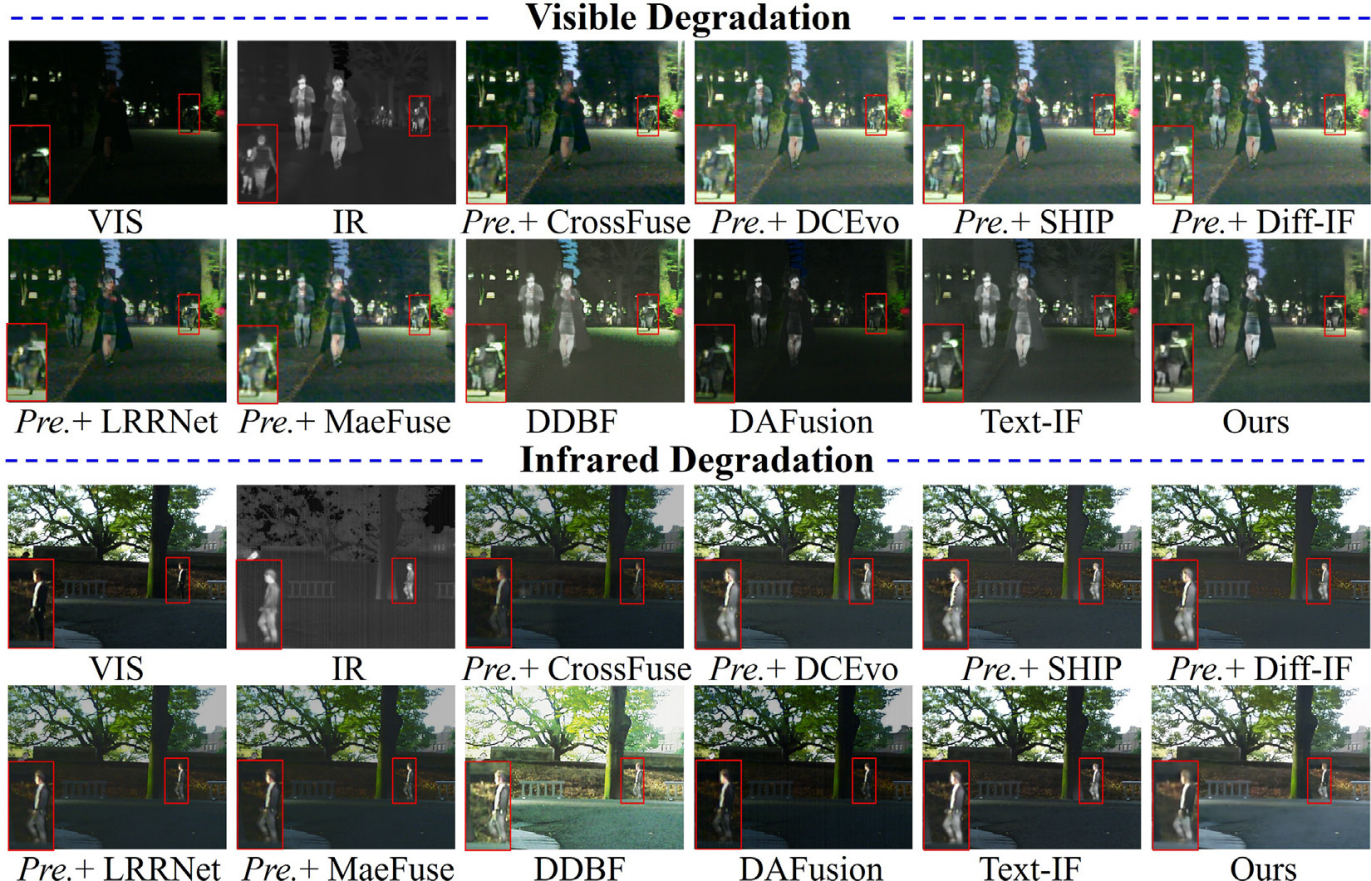
**Qualitative results in infrared degradation and visible degradation scenarios**.

As reported in Table 3, our method consistently achieves the highest scores across multiple evaluation metrics, including MI, Qabf, and PSNR. These quantitative results demonstrate that our model can effectively integrate complementary information even when one source is heavily compromised, maintaining higher structural fidelity compared to other state-of-the-art methods. Qualitative comparisons in Fig. 9 further highlight this superiority. When the visible modality is obscured by darkness, our framework successfully leverages the clean infrared signals to recover distinct pedestrian contours and background textures. Similarly, in cases of infrared blurring, our model preserves sharp edges from the visible modality. This performance is attributed to the proposed robust coding embedding mechanism, which dynamically suppresses interference from the degraded modality while protecting reliable features from the clean one. These results confirm that our approach effectively prevents modal dominance and structural collapse, ensuring strong practical applicability in complex, real-world environments.

**Table 3:** **Quantitative results under infrared degradation or visible degradation scenarios**.

#### 4.4.2 Multi-type and multi-level degradations

To further evaluate the robustness of the proposed method under challenging degradation scenarios, additional experiments were conducted by introducing various degradation types and intensity levels to the infrared modality, while keeping the visible modality unchanged. Specifically, three representative degradation types were considered, including noise, low contrast, and stripe artifacts. For each degradation type, three intensity levels were applied, denoted as S, M, and L, corresponding to small, medium, and large degradation strengths, respectively.

**Fig. 10:**
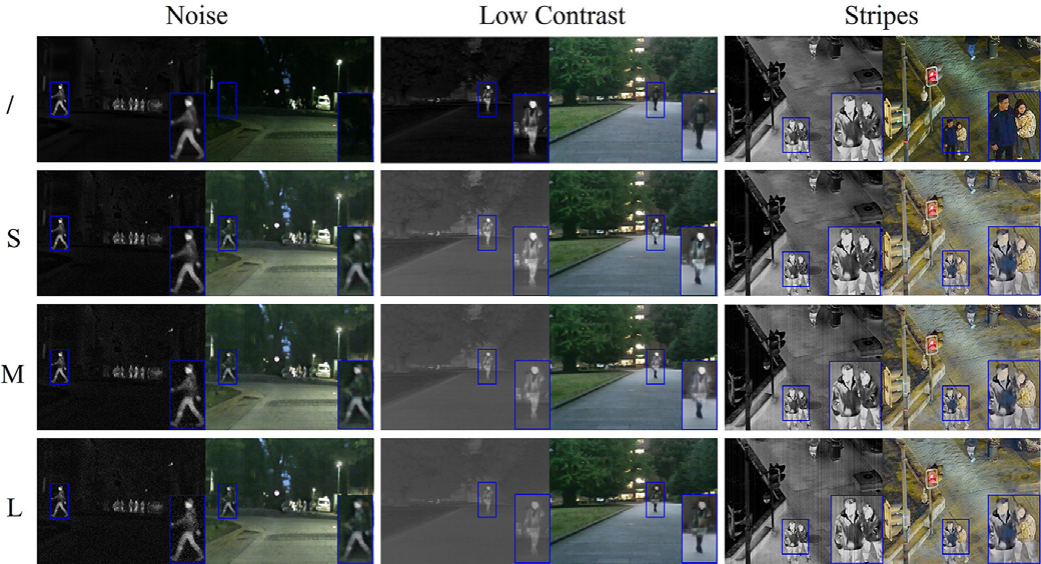
**Qualitative results under different degradation types and intensity levels**.

Fig. 10 illustrates the qualitative results. The first row presents the original infrared and visible inputs, and the subsequent rows show degraded infrared images with their corresponding fused results. As the degradation intensity increases, the infrared inputs exhibit progressively stronger corruption. Nevertheless, the proposed method consistently preserves structural integrity and target saliency across all settings. Even under severe degradation, the fused images effectively suppress artifacts while maintaining thermal targets and visible textures, with stable contrast and structural consistency, demonstrating strong robustness to degradation-related disturbances.

Table 4 reports the quantitative evaluation under all degradation settings. The proposed method maintains stable metric performance across different degradation types and intensity levels, with only marginal fluctuations as degradation strength increases. This demonstrates that the framework is robust to diverse corruption patterns and can consistently produce reliable fusion results under challenging conditions.

**Table 4:** **Quantitative results under different degradation types and intensity levels**.

### 4.5 Ablation study

To further verify the effectiveness and necessity of each module in the proposed method, a series of ablation experiments are conducted on the MFNet dataset, where four model variants were designed for comparison. ***Model I:*** removes the robust embedding module, relying solely on basic latent space modeling. ***Model II:*** eliminates the image-level discriminator, retaining only the feature-level adversarial constraint. ***Model III:*** removes the feature-level discriminator, relying exclusively on the image-level adversarial constraint. ***Model IV:*** excludes the structure-preserving loss and optimizes the model using only the adversarial loss. ***Model V:***the intermediate features $\Omega_{M}^{C_{H}}$ are fused by element-wise maximum selection between the two modalities. ***Model VI:*** a lightweight learnable weighting parameter is introduced to $\Omega_{M}^{C_{H}}$, enabling adaptive re-scaling fusion operation. ***Model VII:*** the robust embedding module is trained using only the feature-level loss, while removing the image-level loss. ***Model VIII:*** The robust embedding module is optimized solely with the image-level loss, without feature-level loss. ***Model IX:*** the entire framework is fine-tuned in an end-to-end manner without freezing the pre-trained autoencoder and robust embedding modules.

**Fig. 11:**
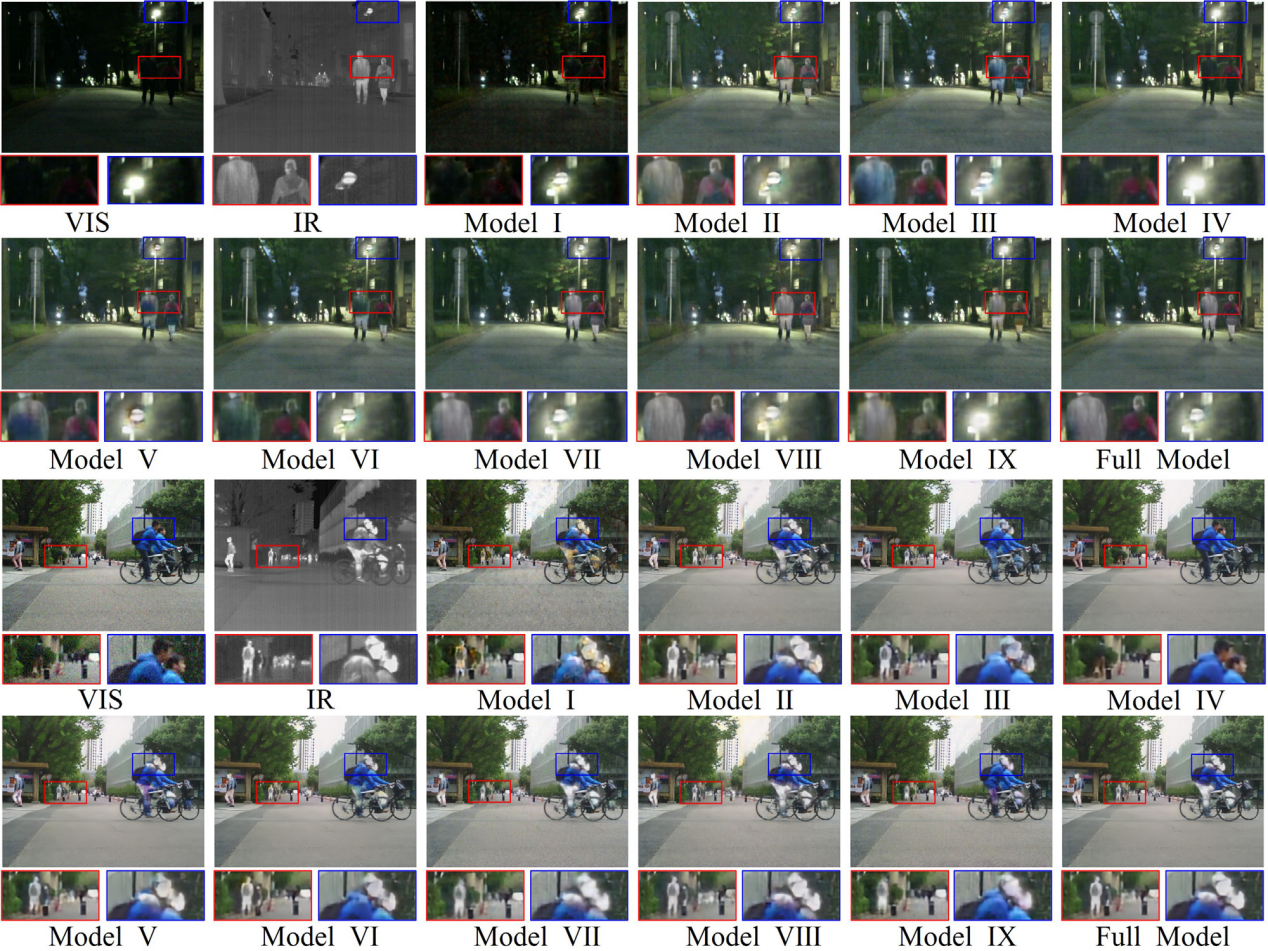
**Qualitative results of the ablation study on the MFNet dataset**.

**Fig. 12:**
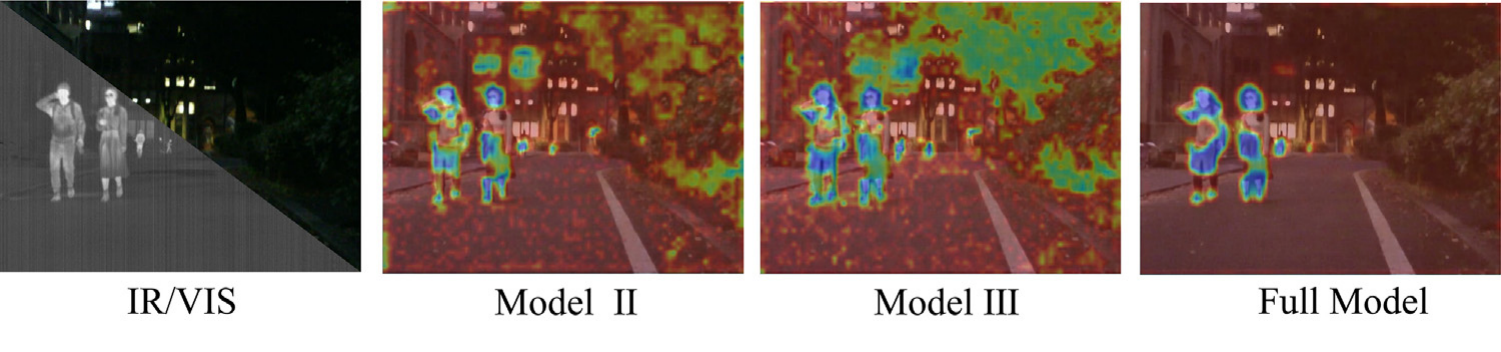
**Visualization of spatial fusion weight maps under dual-space probabilistic adversarial learning**.

The qualitative comparisons are shown in Fig. 11. Removing the robust embedding (Model I) results in noticeable noise and residual degradations, revealing its importance in suppressing interference and stabilizing latent representations. Without the image discriminator (Model II), the fused results exhibit reduced contrast and a grayish tone, indicating weakened global distribution consistency. Eliminating the feature discriminator (Model III) degrades cross-modal alignment and texture preservation. The corresponding fusion weight maps are visualized in Fig. 12. It can be observed that removing either the feature-level or image-level loss results in weight distributions that remain spatially stable, without evident oscillation or contradictory allocation patterns. Compared with these variants, the full model produces clearer modality preference in salient regions while maintaining balanced allocation in background areas. This demonstrates that the dual-space adversarial objectives provide complementary guidance rather than conflicting signals in weight generation. Excluding the structure-preserving loss (Model IV) leads to unstable training and modality bias, highlighting its role in maintaining cross-modal balance. For the intermediate feature fusion strategy, element-wise maximum selection (Model V) causes locally over-enhanced responses and suppresses complementary information, reducing structural coherence. Although introducing a lightweight learnable weighting (Model VI) alleviates this issue to some extent, the lack of explicit cross-modal modeling still limits coordination compared with the full design. Regarding loss and training strategies, retaining only the feature-level loss (Model VII) preserves structural stability but weakens global contrast, while using only the image-level loss (Model VIII) improves perceptual realism at the expense of latent consistency. End-to-end fine-tuning without freezing pre-trained modules (Model IX) results in performance fluctuations under complex degradations, demonstrating the necessity of staged training. Overall, each component contributes to robust encoding, cross-modal alignment, and detail preservation. The complete model achieves the best structural consistency and visual quality, which is further supported by the quantitative results in Table 5.

**Table 5:** **Quantitative results of the ablation study**.

### 4.6 Application to high-level semantic tasks

To further evaluate the semantic representation capability of DPAFuse, we conducted experiments on two distinct downstream tasks: object detection and semantic segmentation.

**Fig. 13:**
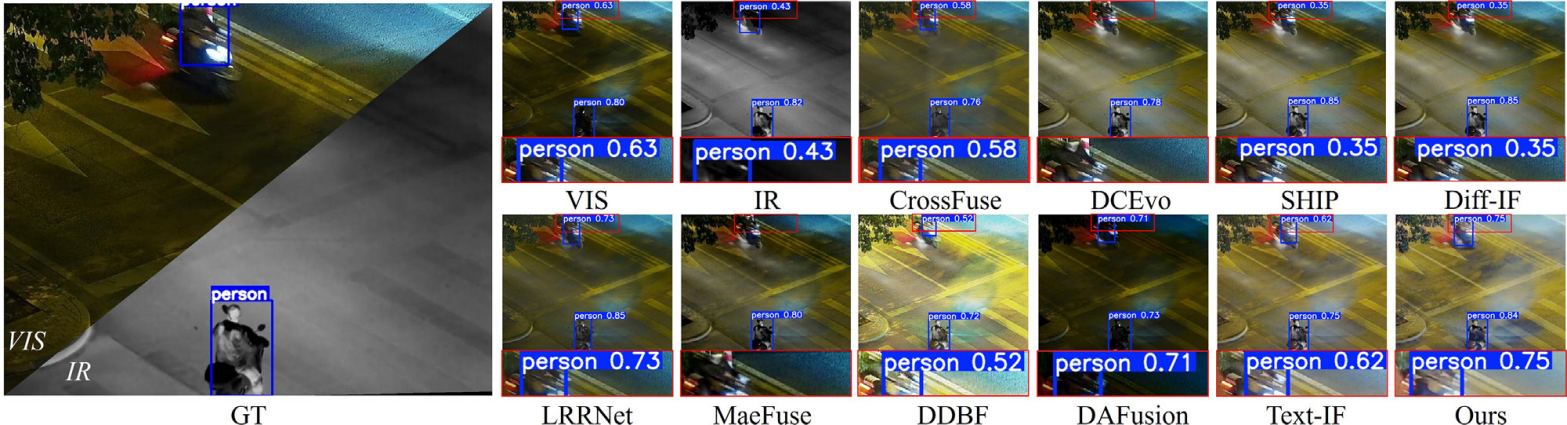
**Qualitative object detection results on the LLVIP dataset**.

1) ***Object detection:*** For this task, we retrained the YOLO-v5 model using infrared images, visible-light images, and the fused images produced by each method to perform pedestrian detection. The experiments were conducted on the LLVIP dataset, with 1000 images for training and 100 images for testing. The quantitative results are reported in Table 6, and the qualitative comparisons are shown in Fig. 13, demonstrating that DPAFuse outperforms the other methods in both detection accuracy and confidence while effectively reducing instances of missed pedestrians.

**Table 6:** **Quantitative object detection results on the LLVIP dataset**.

**Fig. 14:**
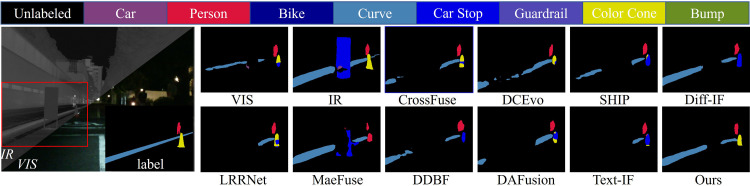
**Qualitative segmentation on the MFNet dataset**.

2) ***Semantic segmentation:*** In the semantic segmentation task, we retrained the SegNext [48] model using infrared images, visible-light images, and the fused images generated by each method. The training and testing sets consist of 784 and 393 images from the MFNet dataset, respectively. Qualitative observations, as shown in Fig. 14, indicate that DPAFuse produces more complete segmentation regions and provides more precise coverage of different target classes. The quantitative results in Table 7 further show that DPAFuse achieves the highest mean Intersection over Union (mIoU).

**Table 7:** **Quantitative segmentation on the MFNet dataset**.

### 4.7 Complexity discussion

We evaluate the parameter counts, floating-point operations (FLOPs), and inference time (measured on 640 × 480 images) of several representative image fusion models to assess their computational burden. The comparison highlights the differences in architectural efficiency across methods and provides a clear understanding of the trade-off between accuracy and complexity. As shown in the Table 8, DPAFuse achieves a favorable balance between computational efficiency and model capacity. Compared with existing fusion networks, our model maintains relatively low computational complexity while preserving strong representational ability. Although it employs a moderate number of parameters, the network demonstrates efficient inference performance and competitive runtime. These findings indicate that DPAFuse achieves an excellent trade-off between efficiency and effectiveness, making it suitable for real-world fusion applications where both speed and accuracy are important.

**Table 8:** **Computational complexity comparison of different fusion models**.

## 5 Conclusion and discussion

This paper addresses the challenge of multimodal image fusion in complex imaging environments, in which the degradations weaken feature representations and the compromise dilemma causes the loss of modality-specific information. To this end, we propose a probabilistic adversarial fusion framework based on robust coding embedding for multimodal images. The proposed method introduces a robust coding embedding mechanism in the latent space, effectively suppressing composite degradation and enhancing cross-modal consistency. Simultaneously, the dual-space probabilistic adversarial fusion mechanism enforces full-link constraints from the feature to the image level, enabling the fused results to comprehensively inherit the complementary advantages of both infrared and visible modalities. Experimental results demonstrate that the proposed method achieves excellent performance across multiple mainstream fusion metrics, particularly outperforming existing approaches in structural consistency, texture fidelity, and target saliency, thereby validating its effectiveness and robustness. In future work, we plan to explore more efficient latent space modeling techniques and extend the framework to video fusion and cross-domain integration of additional modalities, aiming to advance the applications of multimodal perception tasks.

## CRediT authorship contribution statement

**Hao Zhang:** Writing – original draft, Visualization, Validation, Software, Methodology, Funding acquisition, Conceptualization. **Meiqi Gong:** Visualization, Validation, Software, Methodology. **Douyu Wu:** Writing – original draft, Visualization, Validation, Software, Methodology. **Jiayi Ma:** Writing – review & editing, Supervision, Resources, Methodology, Funding acquisition, Conceptualization.

## Declaration of competing interest

The authors declare that they have no conflicts of interest in this work.
